# Efficacy of Lipid Nanoparticle-Loaded Sorafenib Combined with Hepatic Artery Chemoembolization in the Treatment of Primary Hepatocellular Carcinoma Complicated with Microvascular Invasion

**DOI:** 10.1155/2022/4996471

**Published:** 2022-05-20

**Authors:** Wendong Feng, Wendong Cao, Cunwei Cui, Xingtao Pi

**Affiliations:** Department Interventional Therapy, Shanxi Bethune Hospital, Taiyuan, 030000 Shanxi Province, China

## Abstract

This work was to evaluate the therapeutic effect of lipid nanoparticle-loaded sorafenib combined with transcatheter artery chemoembolization (TACE) in patients with primary hepatocellular carcinoma (HC) complicated with microvascular invasion (MVI). In this work, 102 patients with primary HC combined with MVI after radical resection were divided into 4 groups according to different treatment methods. Experimental group 1 was treated with lipid nanoparticle-loaded sorafenib combined with TACE treatment group; experimental group 2 was treated with lipid nanoparticle-loaded sorafenib treatment group; experimental group 3 was TACE treatment group; control group was postoperative routine nursing group. Sorafenib lipid nanoparticles were prepared. The basic information, operation, MVI degree, tumor recurrence, and survival time of patients in each group were recorded and compared to evaluate the therapeutic effect of combined way. No great difference was found in MVI grade, average age, sex ratio, preoperative tumor markers, tumor size, number of patients with liver cirrhosis, operation time, and intraoperative bleeding among the four groups (*P* > 0.05). In addition, the tumor free survival time (TFST), overall survival time (OST), and postoperative 1-year and 2-year survival rates of patients in test group 1 were greatly higher than those in single mode treatment group and control group (*P* < 0.05). In summary, sorafenib nanoparticles combined with TACE can improve the survival status of patients after resection and delay the time of postoperative tumor recurrence.

## 1. Introduction

Primary liver cancer is one of the most common malignant tumors in the world, in which hepatocellular carcinoma (HC) accounts for more than 90% of primary cell carcinoma [1]. According to statistics, there are about 850,000 patients with liver cancer every month all over the world, which is 5% of the total number of cancer patients. The number of deaths of primary liver cancer in the world is as high as 780,000, which is close to one tenth of the total number of cancer deaths. China is the hardest hit area of primary liver cancer. The number and mortality of liver cancer in China are nearly half of the total number of liver cancer cases and deaths in the world. The incidence rate and mortality rate of liver cancer in China are fourth and third [2–5], respectively. When liver cancer is in the early stage, liver transplantation and radical surgical resection are effective, but cancer recurrence often occurs after such treatment. The recurrence rate after liver transplantation can reach more than 30%, while the recurrence rate after resection is very high. Patients with primary HC still have cancer cell metastasis after surgical treatment, resulting in high recurrence. Medical workers cannot improve the long-term survival rate of patients with HC [6–9].

After exploring the causes of recurrence after surgical treatment of primary HC, it is found that the main reason lies in postoperative vascular invasion, which is classified into two: large vascular invasion and microvascular invasion (MVI) [10, 11]. MVI refers to the invasion of tumor tissue into blood vessels, which can usually be diagnosed by preoperative imaging. Macrovascular invasion in patients with HC will increase the risk of cancer recurrence by 15 times. There are three common types of macrovascular invasion in clinic: hepatic vein, portal vein tumor thrombus, and inferior vena cava tumor thrombus [12–14]. MVI refers to the microscopic observation that cancer cell nests invade the vascular lumen of endothelial cells, usually in the portal vein. Microvascular invasion in patients with HC will increase risk of cancer recurrence by about 5 times [15, 16]. Clinically, the occurrence of MVI is confirmed by postoperative case diagnosis. The postoperative MVI usually indicates the recurrence and poor prognosis of patients. MVI is usually divided into three grades according to pathological observation: M0 refers to MVI not found, M1 refers to low-risk group, and namely, there are less than five MVI within 1.0 cm from the tumor. M2 refers to high-risk group; namely, there are more than five MVI within less than 1 cm or MVI beyond 1 cm from the tumor [17–20]. MVI is the influencing factor of postoperative recurrence and metastasis of primary HC. The incidence of MVI in patients with primary HC is high, which is an important factor causing postoperative tumor recurrence and metastasis. Reducing the tumor recurrence rate and prolonging the overall survival time (OST) of patients with primary HC complicated with MVI are widely concerned by medical workers and researchers [21–23].

Transcatheter arterial chemoembolization (TACE) exerts a critical role in the diagnosis and treatment of primary HC and the prevention of tumor recurrence after eradication [24]. The recurrence rate of HC is very high and its prognosis is poor. TACE has the effect of selective hepatic arteriole embolization, which can increase the concentration of chemotherapeutic drugs in liver tissue targets and reduce the damage of drugs to normal liver cells, thereby helping patients to achieve liver function recovery [25]. TACE achieves tumor ischemia and necrosis by embolizing the arteries supplying the tumor. At the same time, a new type of embolization material, drug-loaded microspheres, can also effectively inhibit tumor growth by slow release of chemotherapeutic drugs. Moreover, with the application of microcatheter, superselective segmental hepatic artery embolization can achieve dual embolization of arterial and portal vein, and even some lesions can be cured [26]. However, TACE treatment is a palliative treatment method, and it is difficult to completely inactivate the tumor with a single treatment. According to the data, only 15%-55% of patients will have a local response after TACE treatment, so repeated and multiple treatments are required. In addition, an accurate assessment of the efficacy during the treatment process is required, which is very important for deciding whether to continue the treatment.

As a kinase inhibitor, sorafenib can directly or indirectly inhibit the growth of tumor tissue by inhibiting a variety of signal transduction processes. Various studies have shown that sorafenib can delay the development of the course of advanced HC and prolong the survival time of the patients [27–29]. Lipid nanoparticle is a new type of nanoparticle drug delivery system developed in recent years. The drug is wrapped in the lipid core with solid natural or synthetic lipids to make a solid colloidal particle drug delivery system with a particle size of about 50-1000 nm. It is a nanocarrier prepared with phospholipids as materials. It has a variety of advantages, including high safety, good histocompatibility, and simple preparation engineering. Besides, it can minimize the effect of drugs on cell function [30].

In this study, 102 patients with primary HC who underwent radical resection and were pathologically diagnosed with MVI were divided into four groups based on the treatment willingness of the patients. Experimental group 1: lipid nanoparticles-loaded sorafenib combined with TACE (26 people); experimental group 2: lipid nanoparticle-loaded sorafenib alone (24 people); experimental group 3: TACE alone (25 people). Control group is routine nursing group (27 persons) after operation. Sorafenib lipid nanoparticles were prepared. The basic information, operation, MVI, tumor recurrence, and survival time of patients in each group were recorded and compared to comprehensively evaluate the effect of lipid nanoparticle-loaded sorafenib combined with hepatic artery chemoembolization to treat primary HC complicated with MVI.

## 2. Materials and Methods

### 2.1. Research Objects

102 patients with primary HC who underwent radical resection in our hospital from February 2018 to February 2020 and were pathologically diagnosed with MVI were selected as the research objects. They were rolled into four groups according to different treatment methods. Test group 1 was lipid nanoparticle-loaded sorafenib combined with TACE treatment group (26 people). Test group 2 was simple lipid nanoparticle-loaded sorafenib treatment group (24 people). Test group 3 was simple TACE treatment group (25 people). Control group was postoperative routine nursing group (27 people). This study was approved by the medical ethics committee of the hospital, and all patients and their families signed informed consent.

Inclusion criteria were as follows: (1) patients with primary HC undergoing radical resection; (2) postoperative diagnosis of patients with MVI; (3) aged between 36 and 70 years; (4) patients without other treatment related to this study; (5) patients without other serious diseases; and (6) the patient's family members informed consent to this study.

Exclusion criteria were as follows: (1) patients with other serious diseases; (2) patients who have been treated with similar drugs or TACE; (3) patients unable to complete the study as required; (4) patients with a history of drug allergy; and (5) patients whose family members did not consent and did not sign informed consent.

### 2.2. Preparation and Determination of Lipid Nanoparticle-Sorafenib

#### 2.2.1. Preparation Process

The prescribed amount of sorafenib, glyceryl behenate, and egg yolk lecithin were weighed and dissolved in absolute ethanol to form an organic phase in a water bath at about 80°C. An appropriate amount of poloxamer 188 was taken and dissolved into water at the same temperature. It was kept warm and was formed an aqueous phase. The organic phase was stirred and slowly injected into the aqueous phase with a No. 5 needle. The temperature of the whole process needs to be maintained above the melting point of the lipid material. It was stirred for about 2 hours and concentrated to about 1/2 of the original volume. The obtained translucent system was rapidly dispersed in the aqueous phase at 0-2°C and stirred for 2 hours to obtain a solid lipid nanoparticle suspension.

#### 2.2.2. Precision Measurement

High, medium, and low mass concentrations (100, 50 and 10 *μ*g/mL) with concentrated stock solution were prepared for 5 consecutive injections in the same day and once a day in different days for 5 consecutive days for determination.

#### 2.2.3. Recovery Determination

Three different mass concentrations (100, 50, and 10 *μ*g/mL) were prepared. Three parts of blank lipid nanoparticle suspension were accurately weighed with 1.0 mL each. 1.0 mL of the above prepared sorafenib reference solution of high, medium, and low mass concentrations was accurately added, ultrafiltered, and determined.

### 2.3. Radical Resection

Routine examination shall be performed before operation. General anesthesia shall be performed during operation. The patient was in supine position. The upper abdominal median incision combined with the right transverse incision shall be selected to expose the surgical field of the abdominal cavity and the liver was freed. The position of the internal pipeline structure of the liver was reconfirmed, and the position of the tumor was consistent with the preoperative examination. The first and second hilar were dissected, and the blood flow was blocked in the hilar. Less bleeding was ensured during resection. The edge of the tumor, the relationship with the liver section, the cutting edge, and vessels were reconfirmed. Liver resection with ultrasonic scalpel, electric scalpel, and other instruments was complete.

### 2.4. Postoperative Treatment Process

After resection, different treatment and nursing schemes were implemented for the four groups of patients.

#### 2.4.1. Experimental Group 1

There were 26 people in lipid nanoparticle-loaded sorafenib combined with hepatic artery chemoembolization group. One month after the completion of hepatectomy, the patient was rechecked. If there was no obvious abnormality, the patient was treated with lipid nanoparticle-loaded sorafenib twice a day, 400 mg/time. When the patient had adverse reactions such as abdominal pain, diarrhea, abnormal liver function, nausea, and vomiting, the dosage was reduced to 200 mg/time according to the specific situation. The Seldinger technique was adopted to puncture the femoral artery from the peripheral skin; an appropriate size sheath was placed. Hepatic arteriography was performed. When the angiography results showed no obvious tumor recurrence, preventive TACE treatment was performed. Low-dose quantitative drugs were slowly injected into the artery for treatment. When the angiography results showed tumor recurrence, therapeutic TACE was performed, high-dose chemotherapy drugs were slowly injected into the artery, and TACE treatment was completed every 6 weeks.

#### 2.4.2. Test Group 2

There were 24 persons in sorafenib loaded with lipid nanoparticles alone. One month after the completion of hepatectomy, the patient was rechecked. If there was no obvious abnormality, the patient was treated with lipid nanoparticle-loaded sorafenib twice a day, 400 mg/time. When the patient had adverse reactions such as abdominal pain, diarrhea, abnormal liver function, nausea, and vomiting, the dosage was reduced to 200 mg/time according to the specific situation.

#### 2.4.3. Experimental Group 3

There were 25 persons in simple hepatic artery chemoembolization treatment group. The patients in the TACE group were treated with TACE one month after hepatectomy. Under local anesthesia, the right femoral artery was punctured and intubated with Seldinger technology until the proper hepatic artery. Hepatic arteriography was performed. When the angiography results showed no obvious tumor recurrence, preventive TACE treatment was performed. Low-dose quantitative drugs were slowly injected into the artery for treatment. When the angiography results showed tumor recurrence, therapeutic TACE was performed. High-dose chemotherapy drugs were slowly injected into the artery, and TACE treatment was completed every 6 weeks.

#### 2.4.4. Control Group

There were 27 persons in postoperative routine nursing group. Patients who underwent resection were followed up regularly.

### 2.5. Observation Indicators

#### 2.5.1. Basic Information

The MVI level of patients in each group was statistically recorded: M0 was MVI not found, M1 was the low-risk group, and there were less than five MVI within 1.0 cm from the tumor. M2 was the high-risk group; namely, there were more than five MVI within less than 1 cm or MVI beyond 1 cm from the tumor. The average age, sex ratio, preoperative tumor marker alpha fetoprotein (AFP), tumor size, and liver cirrhosis were recorded and compared.

#### 2.5.2. Operation Time and Intraoperative Bleeding

The average operation time and hand bleeding of patients in each group were observed and recorded during the operation, and then, they were compared between groups. The postoperative TFST and OST of patients were followed up and recorded: TFST referred to the total time from no tumor focus to tumor recurrence and recurrence after radical resection. The OST was the total time from postoperative to death.

### 2.6. Statistical Methods

SPSS software was adopted for data analysis. The data conforming to normal distribution was expressed by mean ± s, and the measurement data was expressed by *t*-test and chi square (*χ*^2^). The test indicated the counting data, and *P* < 0.05 indicated that there was a statistical difference.

## 3. Results

### 3.1. Determination Results of Precision and Recovery of Lipid Nanoparticles

**Figure 1:**
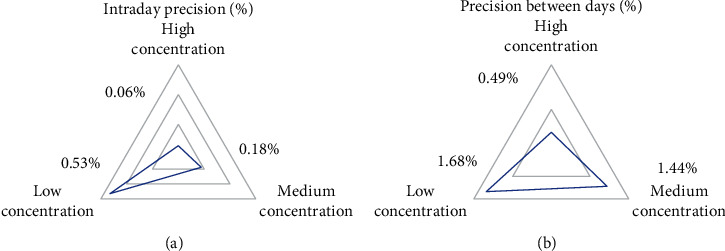
Precision results of sorafenib lipid nanoparticles. (a) showed the intraday precision of lipid nanoparticles. (b) showed the diurnal precision of lipid nanoparticles.

The precision of sorafenib lipid nanoparticles with different concentrations was determined. The results showed that the intraday precision of nanoparticles with different mass concentrations (100, 50, and 10 *μ*g/mL) was 0.06%, 0.18%, and 0.53%, respectively, and the intraday precision was 0.49%, 1.44%, and 1.68%.  Figure 1 gives the specific results.

**Figure 2:**
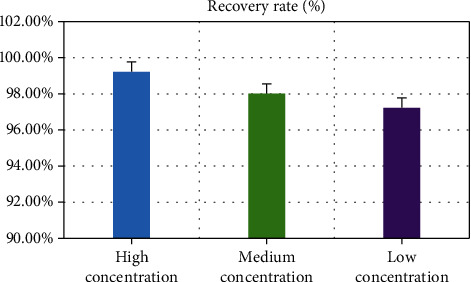
Recovery results of sorafenib lipid nanoparticles.

The recoveries of sorafenib lipid nanoparticles with different concentrations were determined. The results showed that when the mass concentrations were different (100, 50, and 10 *μ*g/mL), the recovery of nanoparticles was 99.22%, 98.01%, and 97.23%.  Figure 2 suggests the specific results.

### 3.2. Comparison of Basic Conditions of Patients

**Figure 3:**
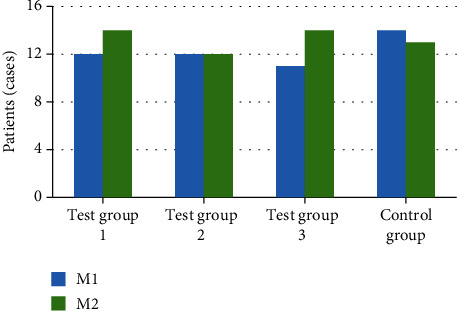
Comparison results of MVI levels of patients in each group.

The number of patients with different MVI levels in each group was compared. The results showed no great difference in the number of patients with M1 and M2 levels in the four groups (*P* > 0.05).  Figure 3 gives the specific results.

**Figure 4:**
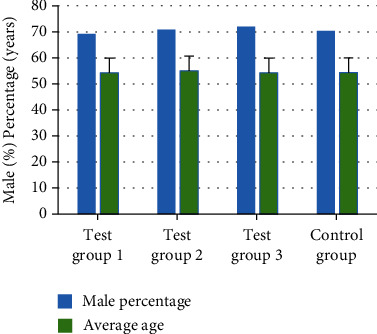
Comparison of male proportion and average age of patients.

**Figure 5:**
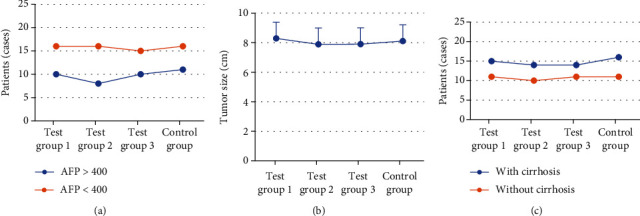
Comparison of preoperative tumor marker AFP, tumor size, and number of patients with liver cirrhosis in each group. Note: (a), (b), and (c) showed the comparisons of preoperative tumor marker AFP, tumor size, and number of patients with liver cirrhosis in the four groups.

The average age, sex ratio, preoperative tumor marker AFP, tumor size, presence or absence of liver cirrhosis, and other basic information of patients in each group were recorded and compared. No obvious difference was found in average age, sex ratio, preoperative tumor marker AFP, tumor size, and number of liver cirrhosis among the four groups (*P* > 0.05). Figures 4 and 5 reveals the specific results.

### 3.3. Comparison of Intraoperative Conditions of Patients

#### 3.3.1. Operation Time and Intraoperative Bleeding Volume

**Figure 6:**
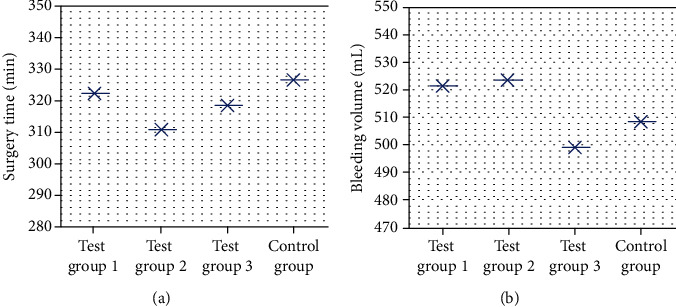
Comparison results of intraoperative conditions of patients in each group. Note: (a) showed the average operation time of four groups of patients. (b) showed the average intraoperative bleeding of the four groups.

The average operation time and hand bleeding volume of patients in each group were observed and recorded during the operation and then were compared between groups. The results show that the operation time of patients in the four groups was within 310-330 min, of which the average operation time of patients in test group 1 was 322.1 min and the average bleeding volume was 521.5 mL. The average operation time of patients in test group 2 was 310.8 min, and the average bleeding volume was 523.5 mL. The average operation time of patients in test group 3 was 318.4 min, and the average bleeding volume was 499.3 mL. The average operation time of patients in control group was 326.2 min, and the average bleeding volume was 508.7 mL. No notable statistical difference was found in the average operation time and intraoperative bleeding volume of patients in different groups (*P* > 0.05).  Figure 6 shows the specific results.

### 3.4. Comparison of TFST of Patients

**Figure 7:**
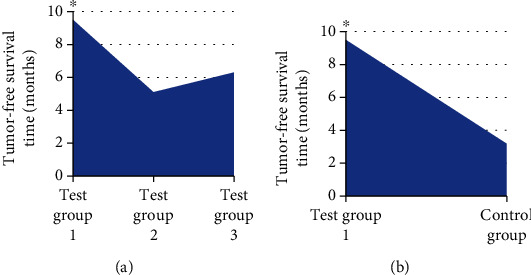
Comparison of TFST of patients in each group. Note: ∗ indicated significant difference: *P* < 0.05. (a) showed the comparison of TFST of patients in test group 1 with single mode treatment. (b) showed the comparison of TFST of patients in test group 1 with routine nursing in control group.

After the operation, the patients in each group were followed up and TFST of the patients was recorded. The results showed that TFST of the patients in the test group 1 was significantly higher in contrast to the single mode treatment group and the control group (*P* < 0.05).  Figure 7 illustrates the specific results.

### 3.5. Comparison of OST and Postoperative Survival Rate

**Figure 8:**
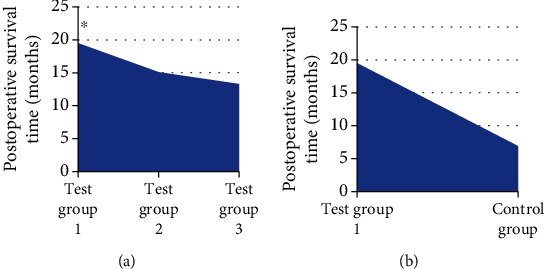
Comparison of postoperative survival time of patients in each group. Note: ∗ indicated obvious difference: *P* < 0.05. (a) showed the comparison of postoperative survival time of patients in test group 1 with single mode treatment. (b) showed the comparison of postoperative survival time of patients in test group 1 with routine nursing in control group.

**Figure 9:**
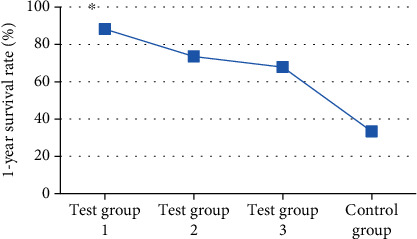
Comparison of 1-year survival time and survival rate of patients in each group. Note: ∗ indicated great difference: *P* < 0.05.

**Figure 10:**
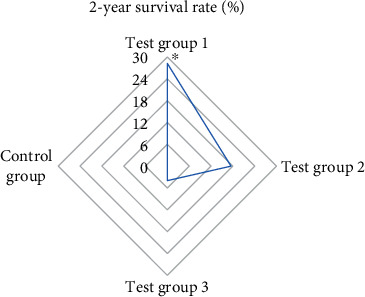
Comparison of 2-year survival time and survival rate of patients in each group. Note: ∗ indicated remarkable difference: *P* < 0.05.

After the treatment, the patients in each group were followed up. The OST of the patients was recorded. The 1-year and 2-year survival rates were calculated. The results showed that the OST of the patients in test group 1 was significantly higher than that in single mode treatment group and control group (*P* < 0.05).  Figure 8 indicates the specific results. The 1-year and 2-year survival rates of patients in test group 1 were observably higher than other groups (*P* < 0.05). Figures 9 and 10 show the specific results.

## 4. Discussion

In clinical treatment of cancer, HC, especially primary HC, has become one of the most common malignant tumors in China. About 850,000 patients suffer from HC in the world every month, which is 5% of the total number of cancer patients in the world, while more than half of the patients with HC are in China [31]. Liver cancer can be classified into HC, cholangiocarcinoma, and mixed hepatocellular and cholangiocarcinoma based on histological type. Among them, primary HC is the most common, more than 90%. Patients with HC have hidden onset, and most of them have no obvious clinical symptoms in the early stage of the disease. Once diagnosed, most patients with HC belong to the middle and late stage, with poor treatment effect and high postoperative recurrence rate [32, 33]. Radical resection and liver transplantation are still the main methods for the treatment of HC, but the high recurrence and metastasis rate seriously affects the prognosis of HC patients. MVI is an independent high-risk factor for postoperative recurrence and metastasis of HC. Therefore, reducing the tumor recurrence rate and prolonging the OST of patients with primary HC complicated with MVI are widely concerned by medical workers and researchers [34]. In this study, 102 patients with primary HC who underwent radical resection and were pathologically diagnosed with MVI were divided into four groups based on the treatment willingness of the patients. Experimental group 1: lipid nanoparticle-loaded sorafenib combined with TACE (26 people), experimental group 2: lipid nanoparticle-loaded sorafenib alone (24 people), experimental group 3: TACE alone (25 people), and control group: routine nursing group (27 persons) after operation. Sorafenib lipid nanoparticles were prepared. The basic information, operation, MVI, tumor recurrence, and survival time of patients in each group were recorded and compared to comprehensively evaluate the effect of lipid nanoparticle-loaded sorafenib combined with hepatic artery chemoembolization to treat primary HC complicated with MVI. Lipid nanoparticles are a new nanoparticle drug delivery system developed in recent years. In this study, the precision of the prepared sorafenib lipid nanoparticles was determined. The results showed that the intraday precision of nanoparticles with different mass concentrations is 0.06%, 0.18% and 0.53%, and the intraday precision is 0.49%, 1.44%, and 1.68%. In addition, it had a high recovery rate, 99.22%, 98.01%, and 97.23%, which could be used in treatment research. The number of M1 and M2 in different grades of MVI in the four groups showed no obvious difference. The comparative analysis of the basic data of all patients in various groups showed that there was no statistical difference in the average age, sex ratio, preoperative tumor marker AFP, tumor size, and the number of patients with liver cirrhosis (*P* > 0.05). The analysis and comparison on average operation time and intraoperative bleeding showed no notable difference (*P* > 0.05), which increased the comparability of the therapeutic effect of lipid nanoparticle-loaded sorafenib combined with hepatic artery chemoembolization. After postoperative treatment of patients in each group, the TFST of patients in test group 1 was much higher than that in single mode treatment group and control group (*P* < 0.05). It showed that the combination of lipid nanoparticle-loaded sorafenib and hepatic artery chemoembolization could delay the postoperative tumor recurrence time of patients with primary HC complicated with MVI. In addition, after the operation, the patients in each group were followed up, the OST of the patients was recorded, and the 1-year and 2-year survival rates were calculated. The results showed that the OST of the patients in the combined treatment group 1 was significantly higher than that in the single mode treatment group and the control group (*P* < 0.05). The 1-year and 2-year survival rates of patients in test group 1 were significantly higher than those in single mode treatment group and control group (*P* < 0.05), which indicated that the combination of lipid nanoparticle-loaded sorafenib and hepatic artery chemoembolization could improve the survival status of patients after resection, prolong the survival time, and improve the survival rate of patients to the greatest extent. Compared with this study, the application of drugs and chemoembolization in the study of Reichert et al. [35] improved the time of disease progression and overall survival rate, which also suggested that the combined treatment had more advantages for patients with primary HC complicated with MVI. In conclusion, the combination of TACE and sorafenib was very important for the prognosis of patients with primary HC complicated with MVI. It could prolong TFST and improve the survival rate.

## 5. Conclusion

In this study, 102 patients with primary HC who underwent radical resection and were diagnosed by pathology with MVI were divided into four groups based on their willingness to treat. Experimental group 1 was lipid nanoparticle-loaded sorafenib combined with TACE treatment group; experimental group 2 was lipid nanoparticle-loaded sorafenib treatment group; experimental group 3 was TACE treatment group; control group was postoperative routine nursing group. Sorafenib lipid nanoparticles were prepared. The basic information, operation, MVI degree, tumor recurrence, and survival time of patients in each group were recorded and compared to comprehensively evaluate the application effect of sorafenib loaded with lipid nanoparticles combined with hepatic artery chemoembolization in the treatment of primary HC complicated with MVI. The results show that the prepared lipid nanoparticles have high precision and recovery rate. The combined treatment of lipid nanoparticle-loaded sorafenib and TACE can delay the postoperative tumor recurrence time of patients with primary HC complicated with MVI, improve the survival status of patients after resection, prolong the survival time, and improve the survival rate of patients to the greatest extent. The deficiency of this study was that the sample size was small and the source was relatively limited. In further research, a large number of patients from different regions and hospitals should be selected, and other combined treatment schemes should be set to explore how to reduce the tumor recurrence rate of patients with primary HC complicated with MVI. In addition, it was aimed at exploring the methods and means to prolong the OST of patients and provide more practical and effective reference value.

## Data Availability

The data used to support the findings of this study are included within the article.
